# House Screening Reduces Exposure to Indoor Host-Seeking and Biting Malaria Vectors: Evidence from Rural South-East Zambia

**DOI:** 10.3390/tropicalmed9010020

**Published:** 2024-01-15

**Authors:** Kochelani Saili, Christiaan de Jager, Freddie Masaninga, Onyango P. Sangoro, Theresia E. Nkya, Likulunga Emmanuel Likulunga, Jacob Chirwa, Busiku Hamainza, Emmanuel Chanda, Ulrike Fillinger, Clifford Maina Mutero

**Affiliations:** 1 International Centre of Insect Physiology and Ecology (icipe), Nairobi P.O. Box 30772-00100, Kenya; psangoro@icipe.org (O.P.S.); theresia.nkya@gmail.com (T.E.N.); ufillinger@gmail.com (U.F.); cmutero@icipe.org (C.M.M.); 2 University of Pretoria Institute for Sustainable Malaria Control, School of Health Systems & Public Health, University of Pretoria, Pretoria 0028, South Africa; tiaan.dejager@up.ac.za; 3 Country Office, World Health Organization, P.O. Box 32346, Lusaka 10101, Zambia; masaningaf@who.int; 4 Mbeya College of Health and Allied Sciences, University of Dar es Salaam, Mbeya 35063, Tanzania; 5 Department of Biological Sciences, University of Zambia, Great East Road Campus, P.O. Box 32379, Lusaka 10101, Zambia; likulungae@yahoo.com; 6 National Malaria Elimination Centre, P.O. Box 32509, Lusaka 10101, Zambia; chirwa.jack@gmail.com (J.C.); bossbusk@gmail.com (B.H.); 7 WHO Regional Office for Africa, Cite du Djoue, Brazzaville P.O. Box 06, Congo

**Keywords:** *Anopheles* mosquitoes, eaves, entomological inoculation rate, sporozoite infectivity rate

## Abstract

This study evaluated the impact of combining house screens with long-lasting insecticidal nets (LLINs) on mosquito host-seeking, resting, and biting behavior. Intervention houses received house screens and LLINs, while control houses received only LLINs. Centre for Disease Control light traps, pyrethrum spray collections and human landing catches were used to assess the densities of indoor and outdoor host-seeking, indoor resting, and biting behavior of malaria vectors in 15 sentinel houses per study arm per sampling method. The protective efficacy of screens and LLINs was estimated through entomological inoculation rates (EIRs). There were 68% fewer indoor host-seeking *Anopheles funestus* (RR = 0.32, 95% CI 0.20–0.51, *p* < 0.05) and 63% fewer *An. arabiensis* (RR = 0.37, 95% CI 0.22–0.61, *p* < 0.05) in screened houses than unscreened houses. There was a significantly higher indoor biting rate for unscreened houses (6.75 bites/person/h [b/p/h]) than for screened houses (0 b/p/h) (χ^2^ = 6.67, df = 1, *p* < 0.05). The estimated indoor EIR in unscreened houses was 2.91 infectious bites/person/six months, higher than that in screened houses (1.88 infectious bites/person/six months). Closing eaves and screening doors and windows has the potential to reduce indoor densities of malaria vectors and malaria transmission.

## 1. Introduction

Malaria is endemic throughout Zambia. In 2021, Zambia’s malaria burden was estimated at 7,050,968 cases, with an incidence rate of 340 cases per thousand per year [1]. The prevalence in children under the age of five years, based on malaria rapid diagnostic tests (RDTs), was found to be 29%, much higher than that recorded in 2018 (9%) [2]. While this increase may reflect the impact of COVID-19 on malaria service delivery [3], it may also indicate a need for additional vector-control methods other than long-lasting insecticidal nets (LLINs) and indoor residual spraying (IRS) [4,5].

The principal vector mosquito species of human malaria, *An. funestus*, *An. gambiae s.s.*, and in some cases, *An. arabiensis,* have a strong preference for feeding on people and resting inside houses [6]. These species are well adapted for entering traditional rural houses using the gaps between walls and roofs (eaves) and may also use open windows and doors to access indoor spaces and blood hosts [7,8]. These behavioral characteristics increase human-vector contact, making these mosquito species efficient malaria vectors [6,9]. More than 80% of human exposure to malaria vectors in sub-Saharan Africa (SSA) is estimated to occur indoors [10]. In southeastern Zambia and Tanzania, approximately 78% of all malaria transmission is estimated to occur indoors [11,12]. Thus, modifying houses to reduce mosquito entry can potentially reduce malaria transmission and provide additional health benefits [5,13]. Such modifications or house improvements include closing eave gaps and screening windows and doors [5,7].

House screening using non-insecticide-treated screens (wire mesh or mosquito netting) as physical barriers on windows and eaves have shown significant protection against malaria [7,14,15,16,17], dengue [18,19], and lymphatic filariasis [20,21]. Despite well-established benefits, house screening has not been encouraged on a large scale by national malaria programs and remains neglected by public health policy. Generating evidence showing the benefits of house screening on vector densities, host-seeking, and biting behavior in specific local settings, particularly under program implementation settings, is thus important. This study evaluated the additive impact of combining house screens with LLINs on mosquito densities and host-seeking and resting behavior. We further evaluated the impact of combining house screens with LLINs on sporozoite infectivity and entomological inoculation rates (EIRs) as a proxy measure of malaria transmission.

## 2. Materials and Methods

### 2.1. Study Area

**Figure 1:**
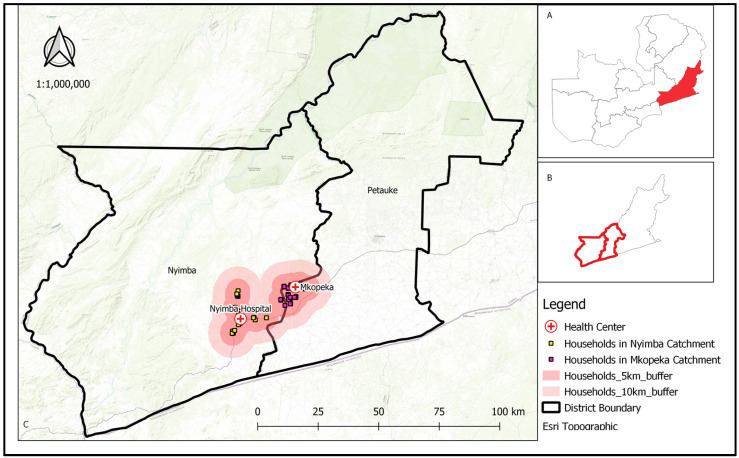
Study area in Nyimba District, Zambia. Insert: (**A**). Map of Zambia showing the location of Nyimba district. (**B**). Location of Nyimba in Eastern province. (**C**). Location of households that were used for entomological collections.

This study was conducted in Nyimba district, located in the Eastern province of Zambia (4°21′0″ S; 30°35′0″ E) (Figure 1). Two neighboring health facility catchment areas were selected for this study: Mkopeka and Nyimba Urban. The study area has been described in detail as part of an entomological baseline study [22] and elsewhere [23,24].

### 2.2. Study Design

This study was a cluster randomized controlled trial using a generalized randomized block design, with the village as the block. This study was part of a larger community-based house screening trial, and the protocol has been reported previously [24]. A total of 89 villages were included in the main study [23].

### 2.3. Community Sensitization and Consent to Participate

Community sensitization meetings with community leaders were held before screens were installed in houses. The chiefs and village headmen were informed about the purposes of the study. Before the installation of screens, voluntary informed verbal consent was obtained.

### 2.4. Study Households, Enumeration, and Participants

**Figure 2:**
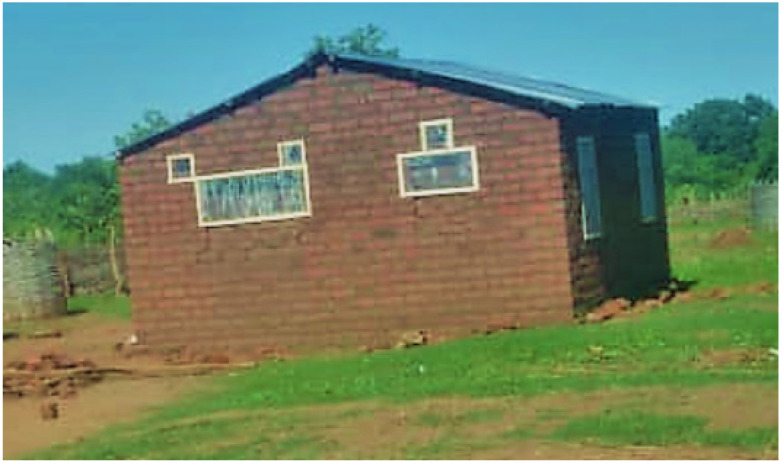
A semi-modern house with firebrick walls and a metal roof showing screened windows and ventilation spaces.

Before sampling and implementing interventions, all households in the two neighboring health facility catchment areas, Nyimba Urban and Mkopeka, were mapped, and household lists were generated. Nyimba Urban is the peri-urban region of the district and is relatively close to the central district administration offices, while Mkopeka is a largely rural region. From the list of households, 800 eligible households were randomly selected. The following inclusion criteria were used: (i) at least two children with ages ranging between 6 months and 13 years; (ii) the house should be semi-modern, defined as a house with a roof made of corrugated iron sheets and with walls that were either mud or fire-burnt bricks (Figure 2); and (iii) houses should not have already had screens.

The 800 households were then randomly assigned to the treatment arm (400 houses to receive screens) or the control arm (400 houses), stratified according to region. From March to April 2019, all 800 houses were provided with at least one LLIN per two persons to ensure optimum coverage of at least one of the primary vector-control interventions as per national guidelines [1]. During the entire study period, no IRS was conducted in the two catchment areas. Routine LLIN distribution continued throughout the study period.

### 2.5. Installation of House Screens

House screens, specifically doors, windows, and ventilation spaces (Figure 2) were installed between December 2019 and January 2020. From the list of houses, screens were installed in 400 randomly selected houses. The remaining 400 houses served as controls. Each catchment area was divided into two zones made up of villages closer to each other. Each zone contained approximately 200 households.

### 2.6. Adult Mosquito Collections

Adult mosquitoes were collected using three different sampling methods: indoor and outdoor Centre for Disease Control ultraviolet light traps (CDC-LTs, Model 512, John W Hock, Gainesville, FL, USA), pyrethrum spray catches (PSCs), and human landing catches (HLCs). Mosquito collections took place after the screens were installed. Mosquitoes were collected in only 20 villages spread across the two study areas. Different sampling methods were used to account for different behaviors.

#### 2.6.1. Light Traps

For each study arm, 15 houses were randomly selected to serve as sentinel houses. Houses were replaced when either consent was withdrawn, or the sleeping structure was destroyed. In that case, the nearest neighbor was used.

On the night of collection, two CDC-LTs per house were deployed: one inside and another outside. The CDC light traps were set from 18:00 to 6:00. Indoors, the CDC-LT was suspended 1.5 m above the floor and approximately 1.5 m away from the feet of a consenting adult sleeping under an LLIN. For outdoor collections, the CDC-LT was hung nearest to where the family would sit to eat and/or spend evenings. Both indoor and outdoor CDC-LTs were not baited. These collections took place once every month between February 2020 and June 2020 and between December 2020 and June 2021, representing 12 collection months.

#### 2.6.2. Indoor Resting Collections

PSCs were conducted using Mortein Energy ball^®^ (Reckitt Benckiser, Alberton, South Africa) as a knockdown spray [25]. PSCs were performed once a month in a second set of 15 sentinel houses randomly selected, eight houses in Mkopeka and seven from Nyimba Urban. Indoor resting collections took place from February 2020 to June 2021, representing 17 collection months. During both CDC-LT and PSC collections, housing characteristics, such as open eaves and the type of material used for wall and floor construction, were recorded.

#### 2.6.3. Human Landing Catches

Species-specific biting behavior and host-searching times were determined using paired indoor and outdoor HLCs in the three villages with the highest indoor mosquito densities, based on PSCs, in the Mkopeka catchment area. Collections took place during two periods: the wet season (April and May 2020) and the dry season (September and October 2020). In each month and from within the three villages, six houses were randomly selected: three control households and three intervention houses. HLCs were conducted for 5 nights, giving an overall of 30 nights per season per study arm. No HLCs were conducted between April and May 2021 due to COVID-19 restrictions in line with the COVID-19 national guidelines of Zambia’s Ministry of Health.

Male volunteers were recruited from a pool of CHWs who had participated in community-based entomological surveillance during the baseline study [22], prior to the intervention installation. All volunteers underwent a 5-day training in basic entomological surveillance including practical sessions on HLCs.

To conduct HLCs, pairs of male volunteers, one indoors and the other outdoors (at least 2 m away from the house), sat with their legs exposed to attract mosquitoes. As mosquitoes attempted to bite, they were collected with a mouth aspirator. Indoor and outdoor collections were conducted between 18:00 and 06:00 in houses occupied by a consenting adult male member of the household sleeping under a mosquito net. Mosquitoes were caught for 45 min each h, allowing a 15 min break.

### 2.7. Species Composition

All collected *Anopheles* mosquitoes were morphologically identified using dichotomous keys [26]. Culicine mosquitoes were only identified at the subfamily level. Members of the *An. gambiae* complex (n = 100) and *An. funestus* group (n = 141) were further identified at the sibling species level by polymerase chain reaction (PCR) [27,28].

### 2.8. Detection of Plasmodium falciparum Infection in Mosquitoes

Sporozoite infectivity was determined for *Anopheles* mosquitoes using sandwich enzyme-linked immunosorbent assays [29,30]. Based on the number available, randomly picked anopheline mosquitoes of the following species were tested for *P. falciparum* circumsporozoite proteins (*Pf* CSPs): *An. funestus* (n = 162), *An. gambiae s.l.* (n = 118), *An. pretoriensis* (n = 109), *An. rufipes* (n = 112), *An. maculipalpis* (n = 47), *An. gibbinsi* (n = 18), and *An. coustani* (n = 2). We heated the ELISA lysates to avoid false CSP positives common in zoophilic species [31].

### 2.9. Data Analysis

All data were analyzed in R version 4.1.0 software [32]. A generalized linear mixed model (GLMM) using the template model builder *(glmmTMB)* package was used to investigate the impact of house screening on indoor and outdoor malaria vector densities. A GLMM was fitted assuming a negative binomial distribution, and “floor type” and “wall type” were selected as random effects and predictor variables as fixed effects. *p* values were derived for each model.

The mean densities of mosquitoes were estimated by dividing the total number of mosquitoes collected by the total number of trapping nights per household. The risk ratio (RR) was used to estimate effect sizes associated with the differences in mosquito densities between screened and unscreened houses. The log risk ratios were transformed into risk ratios (RRs) using “predict” in the R “metafor” package. The modeled percent reduction in mosquito densities in screened houses compared to unscreened houses was calculated as 100 × (1 − RR). All analyses were species-specific. Anopheline mosquitoes were collected in low numbers and pooled for analysis.

To further determine the protective efficacy of the house-screening intervention, the following entomological indices were used: Human biting rate (HBR), defined as the mean number of bites per person per night by a vector species collected either indoors or outdoors. Indoor and outdoor species-specific hourly human biting rates (HBR) were calculated from HLCs. As HLCs were conducted for 45 min within each hour, average bites by mosquitoes were further divided by 0.75 (=45/60 min) to obtain the hourly catch rate. Furthermore, hourly biting rates were categorized into periods as evening (18:00 to 20:45), early night (21:00 to 23:45), midnight (00:00 to 02:45), and early morning (03:00 to 05:45).

The sporozoite infectivity rate (SIR) is defined as the proportion of *Anopheles* mosquitoes with sporozoites in their salivary glands relative to the total number of mosquitoes examined for sporozoites.

To determine the protective efficacy of the house-screening intervention, EIR was used as a measure of malaria transmission. EIR is defined as the number of infectious bites per person per unit of time, usually expressed per year or month.

Due to few mosquitoes being collected by HLCs, species-specific EIR was calculated by multiplying HBR obtained from CDC-LTs (HBR_CDC-LT_) by the SIR. Species-specific HBR from CDC-LTs was calculated as the mean number of female *Anopheles* mosquitoes caught per trap/night without adjusting for room occupancy. Since the CDC-LTs were set only during the wet season (February 2020 to June 2020 and again December 2020 to June 2021) EIR was estimated for the wet season and for an average of six months only.

## 3. Results

Overall, in both the intervention and control houses, we conducted 362 indoor and 287 outdoor CDC-LT collections, 473 resting collections, and 60 HLC collection nights. Less frequent outdoor trap nights for CDC-LTs were due to reduced sampling during the rainy season when heavy rains would interfere with trapping.

### 3.1. Anopheles Species Composition

Overall, using both indoor and outdoor collection methods, a total of 1,972 female anopheline mosquitoes were collected. There was a similar species composition in the two study arms. Nine species were identified based on morphological features: *Anopheles pretoriensis* (31.6%; n = 634), *An. funestus* group (n = 393; 19.9%), *Anopheles maculipalpis* (n = 329; 16.7%), *Anopheles rufipes* (n = 253; 12.8%), *Anopheles gambiae* s.l. (n = 232; 11.8%), *Anopheles coustani* s.l. (n = 68; 3.4%), *Anopheles gibbinsi* (n = 53; 2.7%), *Anopheles squamosus* (n = 13; 0.7%), and *Anopheles. tenebrosus* (n = 7; 0.4%). Additionally, males of the following species were collected: *An. pretoriensis* (n = 13), *An. funestus* (n = 6), *An. rufipes* (n = 5), *An. maculipalpis* (n = 2), and *An. gambiae* s.l. (n = 2). All species, except *An. tenobrosus,* were found in both unscreened houses and screened houses. *An. tenebrosus* was found only in unscreened houses. A total of 644 female culicine mosquitoes were collected.

PCR was performed on a random subsample of 141 (35.9%) collected female *An. funestus* mosquitoes, of which 21 (14.9%) did not amplify. Of the specimens that amplified (n = 120), *An. funestus* s.s. was the dominant species (n = 110; 91.7%). Other species within this group were *Anopheles parensis* (n = 6), *Anopheles leesoni* (n = 2), and *Anopheles rivolurum-like* (n = 2). As *An. funestus* s.s. is the dominant species within this taxon, the *An. funestus* group is henceforth referred to simply as *An. funestus.*

PCR was performed on a random subsample of 100 (43.1%) collected female *An. gambiae* s.l. mosquitoes, of which 15 did not amplify and four gave nonspecific amplifications when further analyzed by ITS2-PCR (n = 2, 500 base pairs; n = 1, 520 bp; n = 1, 600 bp). Of the 81 specimens that were successfully amplified, *An. arabiensis* was the dominant sibling species within the *An. gambiae* complex (n = 61; 75.3%). *Anopheles quadriannulatus* (n = 15) and *An. gambiae s.s.* (n = 5) were the two other species within this complex. As *An. arabiensis* is the dominant species within this complex, *An. gambiae* s.l. is subsequently referred to as *An. arabiensis* throughout this manuscript.

### 3.2. Impact of House Screening on Mosquito Densities

#### 3.2.1. Indoor Host-Seeking

**Figure 3:**
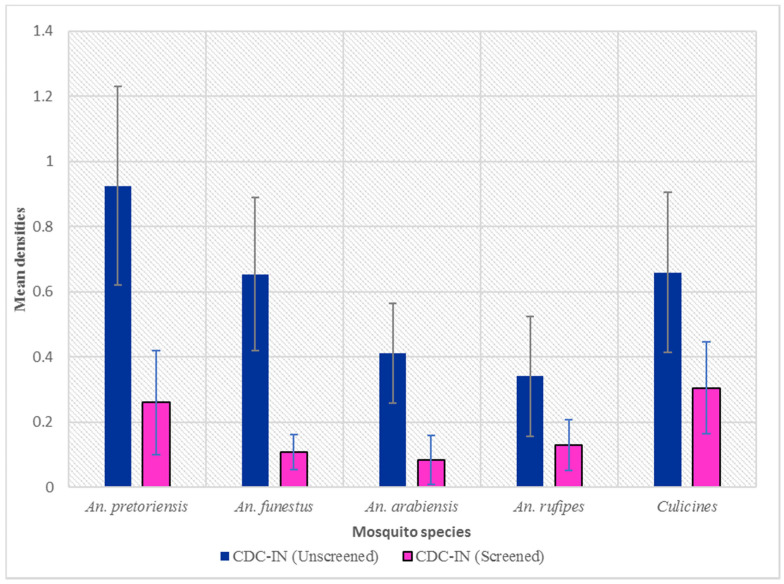
Mean densities of female *Anopheles* and culicine mosquitoes between unscreened (control) and screened (intervention) houses, indoors. Error bars represent 95% confidence intervals. CDC-IN, indoor Centers for Disease Control ultraviolet light traps.

Overall, closing eaves and screening windows and doors significantly reduced the indoor host-seeking densities of *Anopheles* and culicine mosquitoes over two malaria transmission seasons. Based on modeled estimates, overall, there were 44% fewer mosquitoes in screened houses (RR = 0.56, 95% CI 0.43–073, *p* < 0.05) than in unscreened houses. There were 68% fewer *An. funestus* (RR = 0.32, 95% CI 0.20–0.51, *p* < 0.05), 63% fewer *An. arabiensis* (RR = 0.37, 95% CI 0.22–0.61, *p* < 0.05), and 37% fewer *An. pretoriensis* (RR = 0.63, 95% CI 0.46–0.87, *p* < 0.05). Further significant reductions were observed in the indoor host-seeking densities of *An. rufipe*s (RR = 0.61, 95% CI 0.40–0.92, *p* < 0.05), albeit with a small effect size (Figure 3). The densities of culicines were lower in screened houses than in unscreened houses (RR = 0.53, 95% CI 0.41–0.69, *p* > 0.05), although not significantly (Figure 3). No significant reductions (*p* > 0.05) were observed due to screening and closing eaves in the species *An. coustani, An. gibbinsi*, *An. squamosus*, and *An. tenebrosus*, likely due to small sample sizes. Table 1 shows the species-specific mean densities in the control and intervention houses.

#### 3.2.2. Indoor Resting Densities

**Figure 4:**
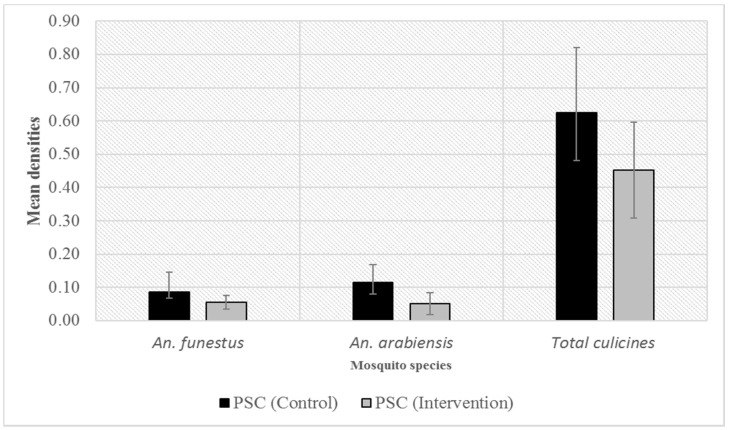
Mean densities of indoor resting female *Anopheles funestus, An. arabiensis* and culicine mosquitoes between unscreened (control) and screened (intervention) houses. Error bars represent 95% confidence intervals.

Overall, closing eaves and screening windows and doors reduced the densities of indoor resting mosquitoes by 20% (RR = 0.80, 95% CI 0.66–0.96, *p >* 0.05) although this was not statistically significant likely due to overall low collections using this method. Considering individual species, reductions in the mean indoor resting density were observed for *An. funestus* (RR = 0.56, 95% CI 0.35–0.91, *p* > 0.05), *An. arabiensis* (RR = 0.61, CI 0.39–0.96, *p* > 0.05), and culicine mosquitoes (RR = 0.65, 95% CI 0.56–0.76, *p >* 0.05) (Figure 4). The species-specific mean densities collected from unscreened and screened houses are shown in Table 2.

### 3.3. Outdoor Host-Seeking

**Figure 5:**
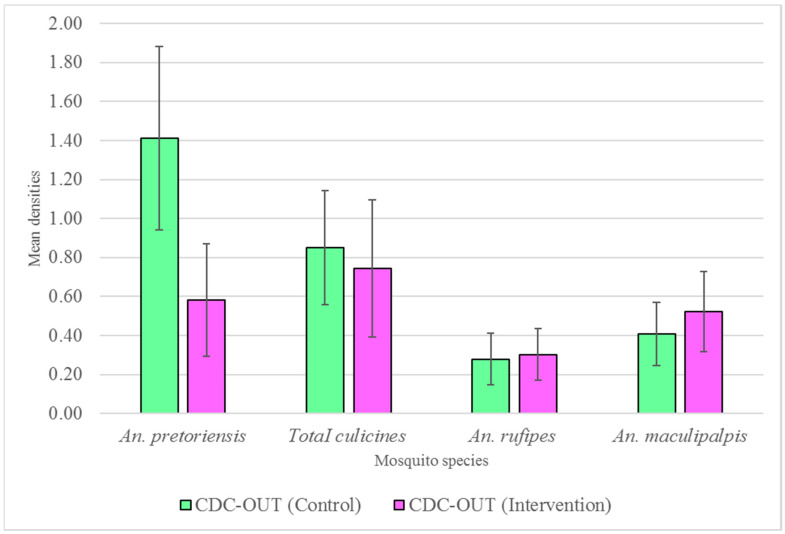
Mean densities of female *Anopheles* and culicine mosquitoes between unscreened (control) and screened (intervention) houses, outdoors using Centers for Disease Control ultraviolet light traps (CDC-LT) placed outdoors (CDC-OUT). Error bars represent 95% confidence intervals.

Overall, outdoor host-seeking (CDC-OUT) mosquito densities were reduced by 27% (RR = 0.73, 95% CI 0.63–0.85, *p* > 0.05) in the intervention group. This reduction was not statistically different. Considering individual species, the most notable and significant reduction was observed in *An. pretoriensis* (RR = 0.60, 95% CI 0.47–0.75, *p* < 0.05). However, more outdoor host-seeking *An. rufipes* and *An. maculipalpis* mosquitoes were collected in the intervention arms than in the control arms, although this was not statistically significantly different (*p* > 0.05). Figure 5 shows the changes in the densities of outdoor host-seeking mosquitoes following house screening. The species-specific mean densities in the control and intervention arms from outdoor host-seeking mosquitoes are shown in Table 3.

### 3.4. Effect of House Screening on Vector Biting Behavior

A total of 51 anopheline mosquitoes were collected using the HLC method during the wet season (April and May 2020), comprising *An. funestus* (n = 25)*, An. arabiensis* (n = 11), *An. maculipalpis* (n = 6), *An*. *rufipes* (n = 4), *An. coustani* (n = 4), and *An. pretoriensis* (n = 1). Since few mosquitoes were collected using this method, pooled results of biting times and rates for all species of anopheline mosquitoes are presented.

No anopheline mosquitoes were collected using HLC in screened houses during the dry season (September and October 2020). A total of five culicine mosquitoes were caught in unscreened houses for the entire dry season. These were discarded with no further analysis provided.

#### 3.4.1. Indoor Biting

**Figure 6:**
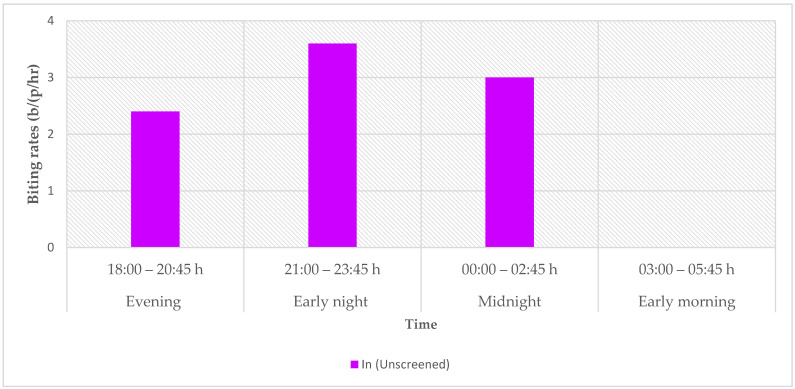
Pooled indoor biting rates for anopheline mosquitoes in unscreened houses for early evening, late evening, early night, and late night.

No mosquitoes were collected indoors in the screened houses. As such, the biting rates for screened houses were not calculated. Thus, there was a significantly higher indoor biting rate for unscreened houses (6.75 bites/person/h [b/p/h]) than for screened houses (0 b/p/h) (χ^2^ = 6.67, df = 1, *p* < 0.05). Pooled results show that the indoor peak biting time was early night, between 21:00 and 23:45, where biting rates were highest at 3.6 b/p/h (Figure 6).

#### 3.4.2. Outdoor Biting

**Figure 7:**
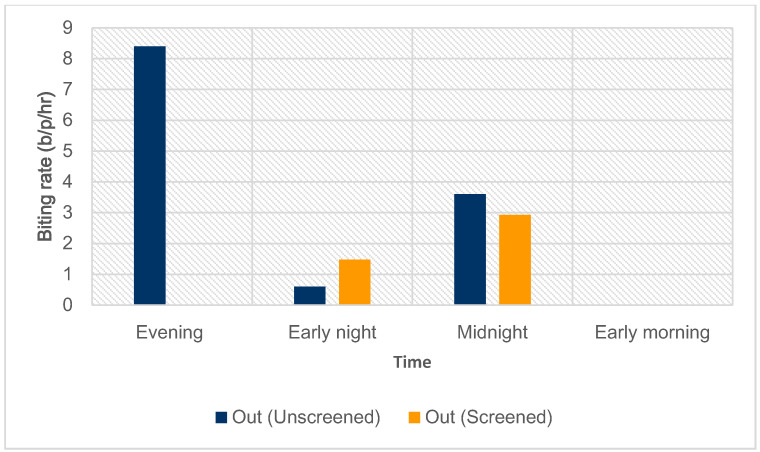
Outdoor biting rates for anopheline mosquitoes in unscreened houses and screened houses (evening 18:00–20:45, early night 21:00–23:45, midnight 00:00–02:45, early morning 03:00–05:45).

Pooled results reveal a higher outdoor biting rate in control houses (9.45 b/p/h) than in intervention houses (3.31 b/p/h) (Figure 7). However, this difference was not significant (χ^2^ = 2.95, df = 1, *p* = 0.08).

The peak outdoor biting time for unscreened houses was evening between 18:00 and 20:45, where biting rates were estimated at 8.4 b/p/h. In screened houses, the peak biting period was at midnight between 00:00 and 02:45.

### 3.5. Sporozoite Infectivity Rates

A total of 102 female *An. funestus* collected indoors were tested for *Pf* CSP. Of these, four tested positive for sporozoites, giving an overall SIR of 3.92%. Of the four sporozoite-infected mosquitoes, two were from unscreened houses, and two were from screened houses. All sporozoite-infected *An. funestus* were trapped between March and May 2020. Sixty female *An. funestus* collected outdoors were analyzed for the presence of *Pf* CSP. Of these, one tested positive, giving an overall sporozoite infectivity of 0.03. The positive *An. funestus* mosquito came from an unscreened house. In all the above, heating the ELISA lysate did not change the *Pf*-CSP positive result.

No other mosquitoes tested positive for sporozoites, giving an overall sporozoite infectivity of zero for both indoors and outdoors for the other species.

### 3.6. Entomological Inoculation Rates

#### 3.6.1. Indoors

Using the indoor biting rates derived from indoor CDC-LT, the indoor EIR for *An. funestus* for unscreened houses was estimated to be 2.91 infectious bites/person/six months during the wet season.

The EIR for *An. funestus* in screened houses was estimated to be 1.88 ib/p/six months. Therefore, the overall estimated indoor EIR for unscreened houses was higher than that of screened houses. However, this was not statistically significant (χ^2^ = 0.22, df = 1, *p* = 0.64).

#### 3.6.2. Outdoors

Outdoor EIR was estimated to be 4.0 ib/p/six months for *An. funestus* for unscreened houses during the wet season. Since there were no sporozoite-infected mosquitoes in the intervention houses trapped outdoors, the estimated EIR was 0 ib/p/two months. Thus, there was a significantly higher outdoor EIR in unscreened houses than in screened houses (χ^2^ = 4.0, df = 1, *p* < 0.05). The results are summarized in Table 4.

## 4. Discussion

This study demonstrated that closing eaves and screening windows and doors with non-insecticide-treated wire mesh reduced the indoor densities of host-seeking, biting, and resting mosquitoes. On average, the densities of indoor host-seeking *Anopheles* mosquitoes were reduced by 44.4%. This reduction was observed across all species but was most notable in the major vectors: *An. funestus* and *An. arabiensis,* where densities were reduced by more than 60%. Our results are consistent with those from Ethiopia [8] and Gambia [17], where 40% and 43% reductions in the mean densities of *An. gambiae* s.l. were observed after house screening. In Kenya, Abongo et al. [14] reported 60% and 54% fewer *An. funestus* and *An. arabiensis* densities after closing eaves and screening houses. In our study, the indoor densities of culicine mosquitoes were also reduced following the screening of eaves, windows, and doors, which is consistent with other studies [8,14,20,21,33]. Screening of houses thus reduces biting from nuisance mosquitoes and protects against viral and parasitic infections [13,20,21].

The reduced densities of mosquitoes likely explain the reduced biting activity of malaria vectors in screened houses. We also observed significantly lower indoor human biting rates in screened houses than in unscreened houses, according to HLCs. PSCs were used to estimate the densities of indoor resting mosquitoes. We collected relatively fewer mosquitoes using PSCs, which may explain the small effect sizes.

In this study, most of the mosquitoes belonged to the *An. funestus* group and *An. gambiae* complex, with most being *An. funestus* and *An. arabiensis,* respectively. This is consistent with previous reports from the area [22]. *An. funestus* is largely anthropophilic and endophilic [6]. *An. arabiensis,* on the other hand, exhibits a wider range of feeding and resting behavior and is able to feed on humans indoors and escape to rest outdoors [6]. Furthermore, in our study, only *An. funestus* tested positive for *Pf* sporozoites. This supports evidence that house screening may have the greatest impact on anthropophilic, endophagic, and/or endophilic species [7], which are also the most efficient malaria vectors [6,34].

To determine the protective efficacy of the house-screening intervention, we used EIR as a proxy measure of malaria transmission [35,36]. Although not significantly different, we estimated that people living in screened houses would receive fewer infectious bites per person (1.88 ib/p) than those living in unscreened houses (2.91 ib/p) during the wet season. These results are similar to those reported in Ethiopia [15] and Tanzania [16]. The likely explanation for the moderate efficacy of house screening experienced in this study could be that residents may have left the doors open, allowing mosquitoes to enter. The door screens installed in our study were not self-closing, an addition recommended for future studies. Second, some door screens were damaged (Saili et al. unpublished), allowing mosquitoes to enter, which was also observed in Gambian [37] and Ethiopian [15] studies.

In this study, EIR was estimated based on human biting rates that were derived from CDC-LTs. HLCs are considered the “gold standard” for collecting human-biting mosquitoes and measuring human-vector contact [38]. Other than the ethical issues [38], HLCs require close supervision and depend on the skill, motivation, and attractiveness of the volunteers collecting the mosquitoes [39]. HLCs may also introduce a mental bias due to the perception that there should be few or no mosquitoes due to an intervention. In this study, fewer mosquitoes were collected using HLCs than when using CDC-LTs, despite collections taking place during the peak malaria transmission season.

Behavioral adaptations of adult mosquitoes, such as feeding and resting outdoors, may limit the effectiveness of house screening on malaria transmission. In contrast, we observed reduced outdoor densities and EIRs in screened houses (4 ib/p/six months in unscreened houses versus 0 ib/p/six months in screened houses). These results are consistent with findings reported in Tanzania [16] and Kenya [14]. Our findings demonstrated slight density reductions for all outdoor species except for *An. maculipalpis.* While house screening primarily affects indoor, human-seeking mosquitoes [15,17], it is noteworthy that the densities of outdoor host-seeking mosquitoes were affected. We postulate that once entry into houses is denied, bloodthirsty endophilic mosquitoes simply seek alternative households or experience population decline due to limited feeding opportunities. However, other factors could have been at play in influencing the densities of mosquitoes outdoors. These may include weather (temperature, humidity, wind speed, rainfall), light levels (moonlight and artificial light), and the presence of domestic animals. Thus, house screening should not be considered in isolation.

*An. pretoriensis* was the most abundant species in our study. *An. pretoriensis* is known to be largely zoophilic and exophagic [40]. Its propensity to forage and rest indoors in this study cannot be entirely explained. Although previous reports from the study area show this species to be infectious [41], no sporozoite-positive infected specimens were found in this study. This was also true for *An. rufipes, An. coustani, An. squamosus, An. maculipalpis,* and *An. gibbinsi*. Thus, despite their abundance, the role of these anopheline mosquitoes in malaria transmission appeared limited during the study period.

This study had several limitations. First, due to logistical challenges and resource limitations, the initial number of households targeted in the original study protocol [24] was not achieved. We experienced a large loss of CDC-LT batteries in the year before collections took place after the screens were installed. The batteries could not be replaced within the study period. We thus acknowledge that the frequency and geographical scope of sampling was not extensive and may explain some of the low vector densities observed in this study. The low numbers could also be attributed to seasonal effects on the productivity of mosquito breeding habitats. This warrants further research in the study area since studies on larval habitat productivity were outside the scope of the present study. A second limitation was the lack of routine (biweekly or monthly) monitoring for holes, rust, or detached screens. This would provide information on the longevity of the screens and indicate the cost-effectiveness of the intervention. This is recommended for future studies. Nonetheless, this study provides evidence that this integrated vector-control approach is effective against malaria vectors, nuisance mosquitoes, and other biting flies and may reduce malaria transmission and other mosquito-borne diseases. Currently, there is growing concern over insecticide resistance [42] and behavioral adaptations of primary malaria vectors to avoid LLINs and IRS [43]. Therefore, mainstream malaria vector-control interventions, namely, IRS and LLINs, which rely on the use of insecticides, may not achieve malaria elimination [5]. Augmenting these core vector-control interventions with supplementary vector-control tools, including house screening, is recommended [3].

## 5. Conclusions

Housing modifications, including closing eaves and screening doors and windows with non-insecticide-treated netting, reduced the indoor density of malaria vectors, including, *An. funestus*, *An. arabiensis,* and culicine mosquitoes. Our findings suggest that house screening has the potential to reduce malaria incidence, prevent diseases, and provide additional benefits, including fewer nuisance bites.

## Figures and Tables

**Table 1:** Species-specific mean densities of indoor host-seeking mosquitoes *.

Species	Unscreened Houses (Control)		Screened Houses (Intervention)	
	N	Mean (95% CI)	N	Mean (95% CI)
*An. funestus*	121	0.65 (0.42–0.89)	19	0.11 (0.05–0.16)
*An. arabiensis*	76	0.41 (0.26–0.56)	15	0.08 (0.01–0.16)
*An. pretoriensis*	171	0.92 (0.62–1.23)	46	0.26 (0.10–0.42)
*An. rufipes*	63	0.34 (0.16–0.52)	23	0.13 (0.05–0.21)
*An. maculipalapis*	43	0.23 (0.12–0.34)	43	0.24 (0.14–0.35)
*An. coustani*	27	0.15 0.03–0.26)	5	0.03 (0–0.05)
*An. gibbinsi*	13	0.07 (0.02–0.12)	8	0.05 (0–0.01)
*An. squamosus*	8	0.04 (0–0.09)	2	0.01 (0–0.03)
Total *Anopheles*	522	2.82	161	0.91
Total Culicines	111	0.6	48	0.27

* Species-specific indoor densities between unscreened houses (LLINs only) and screened houses (LLINs + house screening) based on 185 and 173 indoor CDC-LT trap nights, respectively.

**Table 2:** Species-specific mean reduction in indoor resting mosquitoes *.

Species	Unscreened Houses (Control)		Screened (Intervention)	
	N	Mean (95% CI)	N	Mean (95% CI)
*An. arabiensis*	19	0.09 (0.04–0.13)	14	0.06 (0.02–0.09)
*An. funestus*	25	0.11 (0.06–0.17)	13	0.05 (0.02–0.08)
*An. gibbinsi*	10	0.05 (0.03–0.07)	7	0.03 (0–0.05)
*An. rufipes*	46	0.21 (0.12–0.30)	34	0.13 (0.07–0.20)
*An. coustani*	7	0.03 (0.01–0.06)	5	0.02 (0–0.04)
*An. maculipalapis*	56	0.26 (0.15–0.36)	49	0.19 (0.11–0.28)
*An. pretoriensis*	45	0.21 (0.12–0.30)	66	0.26 (0.15–0.37)
Total *Anopheles*	208	0.95	188	0.75
Total Culicines	135	0.62	48	0.19

* Species-specific indoor resting densities in unscreened (LLINs only) and screened houses (LLINs + House screening) based on 219 and 252 PSC night collections, respectively.

**Table 3:** Mosquito species-specific mean reduction in outdoor host-seeking mosquitoes (CDC-LT OUT) * in Nyimba district, Eastern province, Zambia.

Species	Unscreened (Control)		Screened (Intervention)	
	N	Mean (95% CI)	N	Mean (95% CI)
*An. pretoriensis*	219	1.41 (0.94–1.88)	77	0.55 (0.30–0.87)
*An. funestus*	141	0.91 (0.60–1.22)	54	0.41 (0.20–0.62)
*An. coustani*	15	0.1 (0.04–0.15)	7	0.05 (0.01–0.10)
*An. arabiensis*	59	0.38 (0.22–0.54)	39	0.30 (0.15–0.45)
*An. gibbinsi*	11	0.07 (0.01–0.13)	9	0.07 (0.02–0.12)
*An. rufipes*	43	0.28 (0.15–0.41)	40	0.30 (0.17–0.44)
*An. maculipalapis*	63	0.41 (0.25–0.57)	69	0.52 (0.32–0.73)
Total *Anopheles*	551	3.56	296	2.24
Total Culicines	132	0.85	48	0.36

* Species-specific outdoor densities between unscreened and screened study arms based on 155 and 133 outdoor CDC-LT trap nights, respectively.

**Table 4:** Sporozoite infectivity, indoor and outdoor entomological inoculation rates (EIR) for *An. funestus* and *An. arabiensis* in intervention and control houses in the Nyimba district.

Trap Location	Treatment	Species	# Assayed	# CSP Positive	Sporozoite Rate	Human Biting Rates ^1^	EIR (ib/p/y)
Indoors	Unscreened	*An. funestus*	81	2	0.02	0.65	2.91
		*An. arabiensis*	66	0	0.00	0.25	0.00
	Screened	*An. funestus*	21	2	0.10	0.11	1.88
		*An arabiensis*	19	0	0.00	0.06	0.00
Outdoors	Unscreened	*An. funestus*	40	1	0.03	0.91	4.09
		*An. arabiensis*	22	0	0.00	0.25	0.00
	Screened	*An. funestus*	20	0	0.00	0.42	0.00
		*An arabiensis*	16	0	0.00	0.30	0.00

^1^ Human biting rates were derived from CDC-LTs.

## Data Availability

The datasets used and/or analyzed during the current study are available from the corresponding author upon reasonable request.
